# Corticotropin-releasing factor overexpression in mice abrogates sex differences in body weight, visceral fat, and food intake response to a fast and alters levels of feeding regulatory hormones

**DOI:** 10.1186/s13293-016-0122-6

**Published:** 2017-01-13

**Authors:** Lixin Wang, Miriam Goebel-Stengel, Pu-Qing Yuan, Andreas Stengel, Yvette Taché

**Affiliations:** 1 CURE/Digestive Diseases Research Center and Center for Neurobiology of Stress, Department of Medicine, Digestive Diseases Division, David Geffen School of Medicine, University of California at Los Angeles and Veterans Affairs Greater Los Angeles Healthcare System, Los Angeles, California USA; 2 Present address: Department for Internal Medicine, Martin-Luther-Krankenhaus, Caspar-Theyß-Str. 27-31, 14193 Berlin, Germany; 3 Present address: Department for Psychosomatic Medicine, Charité Berlin, Hindenburgdamm 30, 12203 Berlin, Germany

**Keywords:** Adrenals, Corticosterone, CRF-overexpressing mice, Fasting, Ghrelin, Glucose, Hypothalamic neuropeptide Y, Insulin, Leptin, Sex difference, Visceral fat

## Abstract

**Background:**

Corticotropin-releasing factor overexpressing (CRF-OE) male mice showed an inhibited feeding response to a fast, and lower plasma acyl ghrelin and Fos expression in the arcuate nucleus compared to wild-type (WT) mice. We investigated whether hormones and hypothalamic feeding signals are impaired in CRF-OE mice and the influence of sex.

**Methods:**

Male and female CRF-OE mice and WT littermates (4–6 months old) fed *ad libitum *or overnight fasted were assessed for body, adrenal glands and perigonadal fat weights, food intake, plasma hormones, blood glucose, and mRNA hypothalamic signals.

**Results:**

Under fed conditions, compared to WT, CRF-OE mice have increased adrenal glands and perigonadal fat weight, plasma corticosterone, leptin and insulin, and hypothalamic leptin receptor and decreased plasma acyl ghrelin. Compared to male, female WT mice have lower body and perigonadal fat and plasma leptin but higher adrenal glands weights. CRF-OE mice lost these sex differences except for the adrenals. Male CRF-OE and WT mice did not differ in hypothalamic expression of neuropeptide Y (NPY) and proopiomelanocortin (POMC), while female CRF-OE compared to female WT and male CRF-OE had higher NPY mRNA levels. After fasting, female WT mice lost more body weight and ate more food than male WT, while CRF-OE mice had reduced body weight loss and inhibited food intake without sex difference. In male WT mice, fasting reduced plasma insulin and leptin and increased acyl ghrelin and corticosterone while female WT showed only a rise in corticosterone. In CRF-OE mice, fasting reduced insulin while leptin, acyl ghrelin and corticosterone were unchanged with no sex difference. Fasting blood glucose was higher in CRF-OE with female > male. In WT mice, fasting increased hypothalamic NPY expression in both sexes and decreased POMC only in males, while in CRF-OE mice, NPY did not change, and POMC decreased in males and increased in females.

**Conclusions:**

These data indicate that CRF-OE mice have abnormal basal and fasting circulating hormones and hypothalamic feeding-related signals. CRF-OE also abolishes the sex difference in body weight, abdominal fat, and fasting-induced feeding and changes in plasma levels of leptin and acyl ghrelin.

**Electronic supplementary material:**

The online version of this article (doi:10.1186/s13293-016-0122-6) contains supplementary material, which is available to authorized users.

## Background

Corticotropin-releasing factor overexpressing (CRF-OE) mice have chronic elevation of CRF mainly in the brain regions that normally express CRF [1, 2]. These transgenic mice display Cushing-like features [1, 3, 4], namely elevated plasma levels of the adrenocorticotropic hormone (ACTH) and corticosterone, truncal obesity, muscle wasting, thinner skin, hair loss, immunosuppressive phenotype and insulin resistance linked with the chronic activation of the hypothalamic-pituitary-adrenal (HPA) axis. In addition, CRF-OE mice recapitulate key behavioral and autonomic features of chronic stress which result from CRF overdrive within the brain. These include anti-sexual and anxiogenic behaviors [5–7], reduced attention [8], brain atrophy [9], exaggerated defecation and voiding response to novel acute environment stress [10], increased body temperature and heart rate [2], and reduced fertility in females [1]. We previously reported that female CRF-OE mice also showed a more sensitive pelvic visceral response to a novel environment than males [10]. Further studies by Valentino et al. [11] established that CRF-OE mice display a sex difference in CRF receptor 1 trafficking in the locus coeruleus noradrenergic neurons rendering females more sensitive to high levels of CRF [12]. Therefore, transgenic CRF-OE mice with CRF overdrive in the brain—while showing a phenotype similarly to Cushing’s syndrome—serve also as a relevant model to gain insight in the functional and behavioral consequences of chronic exposure to stress [2, 13–15] and to assess sex differences under these conditions [10, 12]. It is to note that CRF-OE mice have differential etiology of hyperglucocorticoidemia compared to that induced in Cushing’s disease which usually results from tumors in the pituitary or adrenal glands, and occasionally by ectopic ACTH production [16] with an incidence of three to eight times higher in women than in men [17].

We also found that male CRF-OE mice exposed to overnight food deprivation had a reduced refeeding response compared with wild-type (WT) littermates along with decreased Fos expression in the hypothalamic arcuate nucleus [18], suggestive of an altered homeostatic response to food deprivation. The hypothalamus is one of the major integrative centers regulating food intake and energy balance [19, 20]. In particular, neurons in the arcuate nucleus bearing neuropeptide Y (NPY) and proopiomelanocortin (POMC) integrate signals derived from the adipose tissue such as leptin and circulating peptides released from the endocrine cells of the gastrointestinal tract and the pancreas [20]. There is also evidence that obesity alters the fasting and postprandial levels of gut hormones [21]. Therefore, we hypothesized that chronic brain overexpression of CRF and increased visceral adipose tissue in CRF-OE mice [3, 9, 22] could alter the pattern of peripheral hormones and hypothalamic peptides under basal conditions and their response to a fast. Moreover, taking into account the role of sex hormones in the regulation of energy metabolism and the inhibitory actions of glucocorticoids and CRF on the hypothalamic gonadal axis [7, 23, 24], it was important to delineate potential sex differences under these conditions.

In the present study, we first assessed the weights of body, perigonadal adipose tissue, and adrenal glands under basal and fasting conditions in male and female CRF-OE mice compared to WT littermates and their food intake response to an overnight fast. Then, we determined the blood glucose and plasma levels of corticosterone, leptin, insulin, acylated (acyl) ghrelin, peptide tyrosine tyrosine (PYY), and pancreatic polypeptide (PP) known to regulate food intake and energy balance [25, 26]. We also investigated whether hypothalamic expression levels of NPY, POMC and leptin receptor were altered in CRF-OE mice. Lastly, based on reports that the hypothalamic activation of CRF receptor 2 (CRF_2_) suppresses feeding and that CRF_2_ transcript is decreased in obese rats [27, 28], we assessed whether hypothalamic CRF_2_ expression would be altered by fasting in CRF-OE mice.

## Methods

### Animals

CRF-OE mice were bred by the animal core of the UCLA Center for Neurobiology of Stress (Dr. M. Fanselow, Department of Psychology). The source of transgenic line was as originally detailed [1] using the chimeric CRF transgene composed of the rat CRF gene and the 5′ regulator region replaced by mouse methallothionein-1 promoter microinjected into the male pronucleus of fertilized eggs (C57BL/6 × SJL). Mice were backcrossed over ten generations and genotyped using PCR to amplify DNA from the tail as previously detailed [1]. These mice exhibited CRF overproduction mainly in the brain areas, while neither CRF gene was detected by Northern blot analysis in the peripheral tissues except at low levels in the testes nor CRF peptide in the plasma by radioimmunoassay [1]. Adult male and female CRF-OE mice and WT littermates (4–6 months old) were group housed with free access to water and standard rodent chow (Prolab RMH 2500, 5P14, LabDiet, St. Louis, MO) and maintained under controlled conditions of temperature (20–23 °C), humidity, and lighting with a 12-h light/dark cycle (06:00–18:00 hours). Animal care was conducted in accordance with the United States Public Health Service Guide for the Care and Use of Laboratory Animals. The procedures were approved by the Animal Research Committee at VA Greater Los Angeles Healthcare System (# 04012-06).

#### Fasting and food intake measurements

To minimize the stress of novel environment linked with the assessment of refeeding after a fast, mice were first habituated to overnight individual housing in a standard cage with a wire grid without food deprivation twice at a 3-day interval. The wire grid was used to avoid coprophagia during fasting. Fasting/refeeding experiments were performed by placing male and female CRF-OE mice and WT littermates in the individual cage with wired bottom and removing the food overnight and maintaining free access to water. Body weight was monitored before and after fasting. The access to food after the overnight fast started about 09:00 hours (time zero), and food intake was monitored up to 13:00 hours as detailed in our previous studies [18]. In brief, pre-weighed chow was provided at time 0 and at each subsequent time intervals: 0.5, 1, 2, and 4 h, the remaining food and spillage were removed and replaced by new pre-weighed chow. The amount of food intake was determined by the difference between the values of pre-weighed chow and the weight of chow and spillage at the end of each time period.

#### Blood and tissue collections

Male and female CRF-OE and WT mice were randomly assigned to ad libitum feeding or overnight fasting and deeply anesthetized with an overdose of isoflurane between 9:00 and 11:00 hours. Blood (~0.5 ml) was withdrawn immediately from the heart after thoracotomy and transferred in ice-chilled tubes containing ethylenediaminetetraacetic acid (EDTA, 7.5%, 10 μl/0.5 ml blood; Sigma-Aldrich Co., St. Louis, MO). Protease inhibitors were added (EDTA-free protease inhibitor cocktail tablets: cOmplete™, Mini, Roche Applied Sciences, IN; aprotinin 780 KIU; MP Biomedicals, LLC, Solon, OH). Samples were kept on ice and centrifuged at 3000 rpm at 4 °C for 5 min and plasma samples were stored at −80 °C until hormone assays. Adrenal glands and perigonadal adipose tissue were removed bilaterally and weighed. The hypothalamus was dissected out, frozen on dry ice, and stored at −80 °C until RNA extraction.

#### Hormone assays

Plasma corticosterone levels were determined using radioimmunoassay (07-120102, MP Biomedicals, LLC, Solon, OH). The limit of the assay sensitivity was 7.7 ng/ml and the intra-assay variations were less than 4.4%. Plasma levels of insulin, leptin, acyl ghrelin, PYY, and PP were quantified using the mouse gut hormone panel kit by Luminex xMAP (cat. # MGT-78K; Millipore Corporation, Billerica, MA). All samples were processed in one batch and analyzed with the Luminex 100 (Luminex Corporation, Austin, TX). The intra-assay variations were 6.2% for insulin, 9.5% for leptin, 5.5% for acyl ghrelin, 4.5% for PYY, and 6.7% for PP. This technology allows the simultaneous determination of multiple hormones and cross-reactivity between the antibodies, and any other analytes of this panel is negligible (manufacturer’s information, Millipore).

#### Quantitative real-time PCR

The hypothalamus smaples were homogenized and total RNAs were extracted by the phenol-quanidine thiocyanate-chloroform method (TRIzol®, Invitrogen, Carlsbad, CA) following the manufacturer’s recommended protocol. First-strand oligo-dT primed cDNA was synthesized from total RNA (1 μg) of each sample using the ThermoScript™ RT-PCR system (Invitrogen, Carlsbad, CA). The hypothalamic NPY, POMC, and leptin and CRF_2_ receptors were detected by real-time PCR using the DNA Engine Opticon® 2 Detection System interfaced to the Opticon MONITOR™ analysis software version 2.01 (MJ Research Inc., Waltham, MA) in a 25-μl reaction volume. The primers’ sequences are listed in Table 1. The optimized reaction contained 12.5 μl of SYBR® Premix Ex Taq™ (Perfect Real Time, Takara Mirus Bio Inc., Madison, WI), 0.5 μl of oligonucleotide primers (10 μM), 1 μl of the cDNA synthesis reaction, and 10.5 μl of H_2_O. Thermal conditions were as follows: 95 °C for 4 min, followed by 40 cycles of 95 °C for 5 s and 60 °C for 60 s. The specificity of the amplification reaction was determined by performing a melting curve analysis of the PCR fragments. Data were quantified using the comparative cycle threshold (*C*_t_) method. For each sample, *C*_t_ was normalized to S16 and the final value 2^−ΔΔ*C*t^ was adjusted so that the control had a mean relative mRNA level of 1 as previously described [29].

**Table 1:** Primers’ sequences used in quantitative RT-PCR

Name		Accession number
NPY	Forward: 5′-AGAGATCCAGCCCTGAGACA Reverse: 5′-TTTCATTTCCCATCACCACA	NM_023456
POMC	Forward: 5′- GGCTTGCAAACTCGACCTCT Reverse: 5′- TGACCCATGACGTACTTCCG	NM_008895
Leptin receptor	Forward: 5′-CAGAATGACGCAGGGCTGTA Reverse: 5′- TCTGAAATGGGTTCAGGCTCC	NM_146146.2
CRF_2_ receptor	Forward: 5′-CAGTCCTTCCAGGGTTTCTTTG Reverse: 5′- GGTCACACAGCAGCTGTCTGCTT	AY445512
S16	Forward: 5′-TGCGGTGTGGAGCTCGTGCTTGT Reverse: 5′-GCTACCAGGCCTTTGAGATGGA	GenBank M11408

#### Statistical analysis

Data were analyzed by one-way ANOVA, and interactions between genotypes, feeding status, and sex were analyzed by two-way or three-way ANOVAs followed by Tukey’s post hoc tests. Correlations between hormone levels, blood glucose, and tissue weights were performed by linear regression analyses. Data are expressed as mean ± SEM. *P* < 0.05 was considered significant.

## Results

### Body and perigonadal fat weights and food intake response to overnight fast: influence of sex and CRF-OE genotype

#### Body weight

**Fig. 1:**
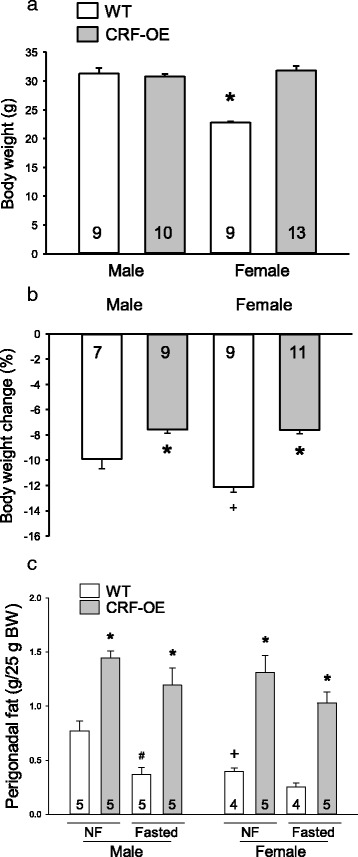
Body weight under normal feeding (**a**) and percentage change after fasting (**b**) and weights of perigonadal fat per 25 g of body weight under basal and after fasting (**c**) of male and female CRF-OE mice and wild-type littermates (*WT*). The number of mice/group is indicated at the *bottom of each bar* in graph. Data are mean ± SEM. *P < 0.05 vs. WT mice of same sex; ^+^ *P* < 0.05 vs. male WT

Under basal conditions (ad libitum feeding), adult 4–6-month-old female WT mice had a significant 28% lower body weight than male WT which was not present in CRF-OE mice (Fig. 1a). Namely, female CRF-OE had a 30% increase in body weight compared to female WT, while male CRF-OE and WT mice had similar weights (Fig. 1a). Two-way ANOVA analysis showed a significant interaction of sex and genotype (*F*_1,35_ = 47.7, *P* < 0.001). After an overnight food deprivation, the body weight was reduced by 9.9 ± 0.8 and 12.1 ± 0.4% in male and female WT, respectively (*P* < 0.01), with a significant sex difference (Fig. 1b). Male and female CRF-OE mice had a similar reduction of their body weight by fasting in both sexes (7.6 ± 0.3 and 7.6 ± 0.3%, respectively; Fig. 1b), and the reduction was significantly lower compared to WT mice of the same sex (*F*_1,32_ = 63.5, *P* < 0.001). Three-way ANOVA showed an impact of sex in WT mice (*F*_1,32_ = 6.8, *P* < 0.05) and an interaction between sex and genotype (*F*_1,32_ = 6.5, *P* < 0.05).

#### Perigonadal fat

Under basal conditions, the perigonadal fat weights expressed per 25 g of body weight were 49% lower in female than in male WT mice, and 88 and 231% higher in male and female CRF-OE, respectively, compared with WT mice of the same sex (Fig. 1c; *P* < 0.001). However, there was no significant difference between male and female CRF-OE mice as observed in the WT group (Fig. 1c). Fasting led to a significant 52% reduction in perigonadal fat weight in WT males while in females, it did not reach statistical significance (Fig. 1c). In male and female CRF-OE mice, the perigonadal tissue weights were not modified by fasting and there was no sex difference (Fig. 1c). Two-way ANOVA showed a highly significant influence of genotypes (*F*_1,47_ = 160.8, *P* < 0.001) and of sex (*F*_1,47_ = 28.3, *P* < 0.001).

#### Food intake

**Fig. 2:**
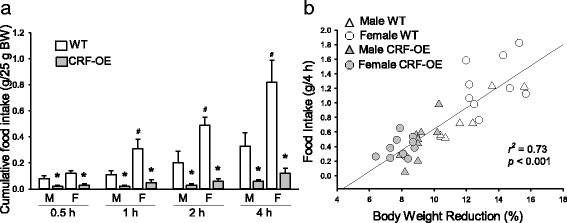
Cumulative food intake after overnight fasting (**a**) and correlation between body weight reduction and 4-h cumulative food intake (**b**) in male and female wild-type (WT) and CRF-OE mice. The number of mice/group is indicated at the *bottom of each bar* in graph **a** and **b**. Data are mean ± SEM, *n* = 4–5/group. *P < 0.05 vs. WT mice of same sex; ^+^ *P* < 0.05 vs. male WT. *M* male, *F* female

The food intake measurements repeated twice in the same cohort of WT and CRF-OE male and female mice showed similar results. The 4-h cumulative feeding response to an overnight fast was 1.5-fold higher in female than in male WT mice calculated per 25 g body weight (*P* < 0.05; Fig. 2a). At 0.5-, 1-, 2-, and 4-h time periods of refeeding, male CRF-OE mice had 75, 82, 88, and 81% lower cumulative food intake than male WT mice, respectively (*P* < 0.05), and similarly, female CRF-OE mice had a 75, 84, 88, and 85% reduction compared to female WT mice (*P* < 0.05; Fig. 2a), respectively. The reduction of food intake was highly correlated with that of body weight (*r* ^2^ = 0.73, *P* < 0.001; Fig. 2b).

### Increased adrenal gland weights with sex difference and plasma levels of corticosterone in CRF-OE mice

**Fig. 3:**
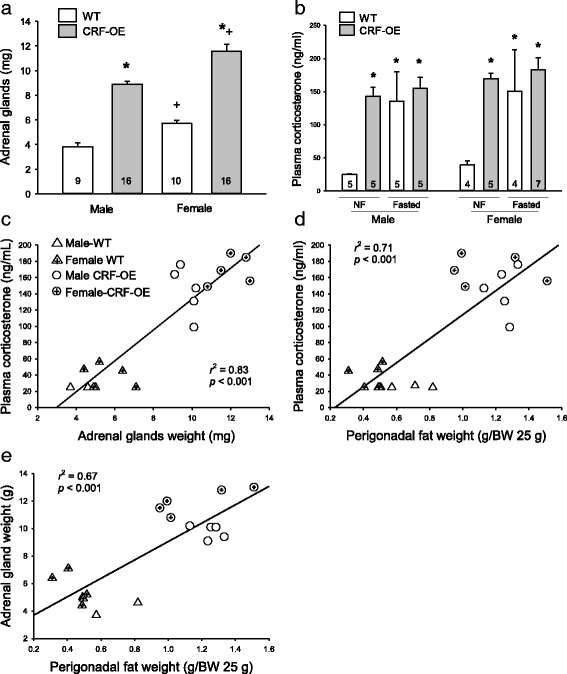
Weights of the adrenal glands (**a**) and plasma levels of corticosterone (**b**) of male and female CRF-OE mice and WT littermates. Data are mean ± SEM. The number of mice/group is indicated at the *bottom of each bar*. *P < 0.05 vs. WT mice of the same sex (vs. non-fasted WT in graph **c**); ^#^ *P* < 0.05 vs. non-fasted male WT mice, and ^+^ *P* < 0.05 vs. male mice of the same genotype. *NF* non-fasted. Correlation between plasma corticosterone levels and weights of adrenal glands (**c**), corticosterone levels and perigonadal fat (**d**), and weights of adrenal glands and perigonadal fat (**e**)

There were no differences in the adrenal glands weights between the left and right sides and between fasted and non-fasted mice (data not shown); thus, the adrenal weights reflect the bilateral pooled values for each mouse and data are expressed in the same genotype and sex group irrespective of feeding status. The larger animal number shown in each group (Fig. 3a) in this assessment reflects data included from previous experiments [9, 18] in which the adrenal glands’ weight was not reported. The weights of the adrenals showed differences in sex and genotypes with larger adrenals in females than in males of both WT (5.7 ± 0.3 vs. 3.8 ± 0.3 mg, respectively; *P* < 0.05) and CRF-OE mice (11.6 ± 0.6 vs. 8.9 ± 0.3 mg, respectively; *P* < 0.001), with values in CRF-OE > WT (*P* < 0.001; Fig. 3a). The significance was attributed to sex (*F*_1,47_ = 28.28, *P* < 0.001) and genotype (*F*_1,47_ = 160.88, *P* < 0.001).

Basal, early light phase, and plasma corticosterone levels were 4.6- and 3.3-fold higher in male and female CRF-OE, respectively, than in WT mice of the same sex under conditions of ad libitum feeding (Fig. 3b). There was no sex difference in both genotypes (two-way ANOVA, sex × genotype, *F*_1,16_ = 0.53, *P* > 0.05). Overnight fasting increased plasma corticosterone in WT mice to the same levels as those in non-fasted CRF-OE mice, while in CRF-OE mice, the plasma corticosterone was not further increased and there was no sex difference in both genotypes (*P* > 0.05; Fig. 3b).

The basal plasma corticosterone levels were highly correlated with the weights of the adrenal glands (*r* ^2^ = 0.83; Fig. 3c) and perigonadal fat (g/25 g body weight (BW)) (*r* ^2^ = 0.71; Fig. 3d), and the latter two were also correlated (*r* ^2^ = 0.67; Fig. 3e).

### Plasma levels of metabolic hormones induced by overnight fast: influence of CRF-OE and sex

#### Leptin (Fig. 4a)

**Fig. 4:**
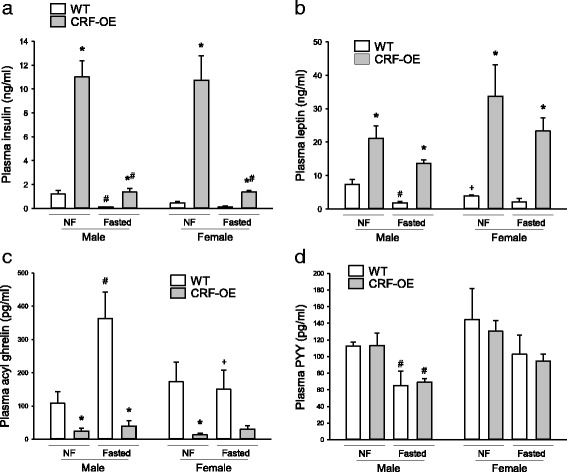
Mouse gut hormone panel assay by Luminex xMAP technology for plasma insulin (**a**), leptin (**b**), acyl ghrelin (**c**), and PYY (**d**) in male and female fasted and non-fasted CRF-OE mice and WT littermates. Data are mean ± SEM, *n* = 4–5/group. *P < 0.05 vs*.* WT mice of same sex under the same feeding status; ^#^ *P* < 0.05 vs*.* non-fasted mice of the genotype; ^+^ *P* < 0.05 vs*.* male mice of the same genotype and feeding status

Basal plasma leptin levels were 0.9-fold lower in female than in male WT mice (*P* < 0.05). Overnight fasting decreased the levels in male WT by 3.2-fold (*P* < 0.05), while they were not significantly modified in female WT mice. By contrast, CRF-OE male and female mice had similarly elevated plasma leptin levels under basal conditions, which were 1.9- and 6.8-fold higher, respectively, than those in WT mice of the same sex (*P* < 0.05), and not significantly altered by an overnight fast in both sexes. Three-way ANOVA showed an influence of genotype, fasting, and an interaction between sex and genotype (Table 2).

**Table 2:** Influence of genotype, fasting, and sex on plasma levels of hormones analyzed by three-way ANOVA (*F*_1,32_)

	Genotype	Fasting	Sex	Genotype × fasting	Genotype × sex	Sex × fasting	Genotype × fasting × sex
Corticosterone	21.99, *P* < 0.001	15.11, *P* < 0.001	n.s.	7.25, *P* < 0.05	n.s.	n.s.	n.s.
Leptin	49.35, *P* < 0.001	5.36, *P* < 0.05	n.s.	n.s.	5.27, *P* < 0.05	n.s.	n.s.
Insulin	82.15, *P* < 0.001	67.19, *P* < 0.001	n.s.	49.93, *P* < 0.001	n.s.	n.s.	n.s.
Acyl ghrelin	42.16, *P* < 0.001	6.14 *P* < 0.05	n.s.	n.s.	n.s.	6.80, *P* < 0.05	6.90, *P* < 0.05
PYY	n.s.	12.55, *P* = 0.001	5.47, *P* < 0.05	n.s.	n.s.	n.s.	n.s.
PP	9.39, *P* < 0.05	n.s.	n.s.	n.s.	n.s.	n.s.	n.s.

*PYY* peptide tyrosine tyrosine, *PP* pancreatic polypeptide, *n.s* not significant

#### Insulin (Fig. 4b)

Under basal conditions, plasma insulin levels in male and female WT mice were low and significantly reduced after an overnight fast by 91.7% in male WT mice (*P* < 0.05) and not significantly in female WT mice. There was no sex difference in both conditions. By contrast, CRF-OE mice had high insulin values (*P* < 0.001) that were similar in males and females and decreased after an overnight fast by 87.6 and 87.2%, respectively; however, the levels were still significantly 12.6- and 8.8-fold higher than those in male and female WT mice, respectively (*P* < 0.05). Three-way ANOVA showed a significant effect of genotype, fasting, and an interaction between feeding and genotypes (Table 2).

#### Acyl ghrelin (Fig. 4c)

Plasma acyl ghrelin levels were not significantly different in male and female WT mice under ad libitum feeding conditions. Overnight fasting resulted in a 2.4-fold increase in plasma acyl ghrelin in male WT mice (*P* < 0.05), while levels did not change in WT females. By contrast, CRF-OE mice compared to WT mice had reduced acyl ghrelin levels (*P* < 0.001) that were similar in males and females under basal and overnight fasted conditions. Three-way ANOVA showed a significant effect induced by genotype, fasting, and an interaction between feeding status and sex and by genotype × sex × feeding status (Table 2).

#### PYY (Fig. 4d)

Plasma PYY levels were similar in male and female WT and CRF-OE mice under basal ad libitum conditions. Overnight fasting significantly reduced PYY values by 0.4-fold only in male mice of both genotypes (*P* < 0.05). Three-way ANOVA showed significant influences induced by fasting and sex (Table 2).

#### PP

No significant change in plasma PP was detected by one-way ANOVA with post hoc comparison among groups as there were large individual differences (Additional file 1: Figure S1), while three-way ANOVA showed a significant impact of genotype, namely CRF-OE vs. WT (Table 2).

#### Correlation between hormones and perigonadal fat

Under basal fed conditions, there was a highly positive correlation between plasma levels of corticosterone and insulin (*r* ^2^ = 0.81, *P* < 0.001) and leptin (*r* ^2^ = 0.45, *P* = 0.001) and a negative correlation with acyl ghrelin (*r* ^2^ = 0.31, *P* < 0.05; Additional file 1: Figure S2). Basal plasma PYY and PP levels were also correlated (*r* ^2^ = 0.62, *P* < 0.001; Additional file 1: Figure S3A). Low correlations were observed between insulin and leptin, insulin and acyl ghrelin, and acyl ghrelin and leptin (*r* ^2^ = 0.25, 0.37, and 0.26, respectively, *P* < 0.05; Additional file 1: Figure S3B–D). Under fasted conditions, plasma levels of leptin and insulin were highly correlated (*r* ^2^ = 0.64, *P* < 0.001, Additional file 1: Figure S4). Other hormones had low or no correlations (data not shown).

The perigonadal fat (g/25 g BW) was correlated with leptin (Additional file 1: Figure S5A) or insulin (Additional file 1: Figure S5B) and inversely with acyl ghrelin (Additional file 1: Figure S5C) both under basal (*r* ^2^ = 0.66, 0.58, and 0.45, respectively, *P* < 0.001) and fasting conditions (*r* ^2^ = 0.52, 0.69, and 0.45, respectively, *P* < 0.001; Additional file 1: Figure S6A–C).

### Hyperglycemia in CRF-OE mice with sex differences

**Fig. 5:**
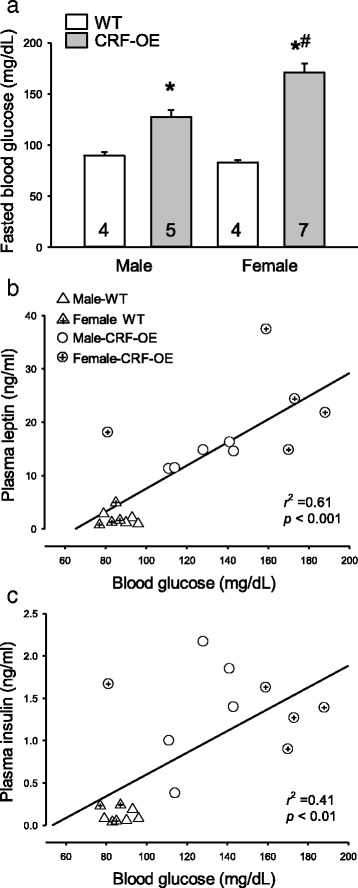
Fasting levels of blood glucose (**a**) and its correlation to fasting plasma leptin (**b**) and insulin (**c**) Data are mean ± SEM. The number of mice/group is indicated in each *bar* of graph **a**. *P < 0.05 vs. WT mice of the same sex and ^#^ *P* < 0.05 vs*.* male CRF-OE mice

After an overnight fast, levels of blood glucose were similar in male and female WT mice (89.5 ± 3.7 vs. 83.0 ± 2.2 mg/dL, *P* > 0.05; Fig. 5a). CRF-OE mice had significantly higher fasting blood glucose levels than WT mice (Fig. 5a) with a sex difference (127.4 ± 6.6 and 170.7 ± 9.0 mg/dL in males and females, respectively, *P* = 0.002). Two-way ANOVA showed significant effects of sex (*F*_1,16_ = 5.67, *P* < 0.05) and genotype (*F*_1,16_ = 66.04, *P* < 0.001) and an interaction between sex and genotype (*F*_1,16_ = 10.39, *P* = 0.005).

Blood glucose levels in fasted CRF-OE and WT mice were positively correlated with plasma levels of leptin (*r* ^2^ = 0.61, *P* < 0.001) and insulin (*r* ^2^ = 0.41, *P* < 0.001; Fig. 5b, c) and, to a smaller extent, negatively with acyl ghrelin (*r* ^2^ = 0.28, *P* < 0.05) and PP (*r* ^2^ = 0.24, *P* < 0.05), but not with plasma PYY (data not shown). Blood glucose was also correlated with body weight and perigonadal fat after fasting (*r* ^2^ = 0.57 and 0.54, respectively, *P* < 0.001).

### Hypothalamic expression levels of NPY and POMC and leptin receptor: influence of fasting and genotype

#### NPY mRNA (Fig. 6a)

**Fig. 6:**
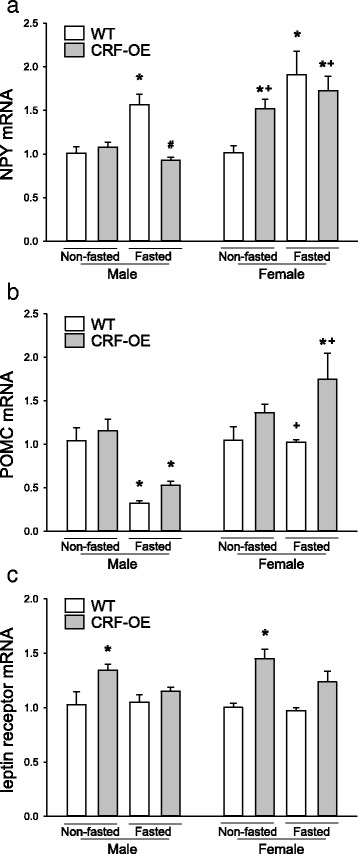
Hypothalamic transcript levels of NPY (**a**), POMC (**b**), and leptin receptor (**c**) in male and female CRF-OE mice and WT littermates under basal or fasted conditions detected by quantitative real-time PCR. The number of mice/group is indicated in each bar. Data are mean ± SEM, *n* = 4–5/group. *P < 0.05 vs. non-fasted WT mice

In mice fed ad libitum, hypothalamic NPY mRNA levels detected by real-time PCR were similar in WT of both sexes and male CRF-OE mice. Overnight fasting significantly increased NPY mRNA expression by 55.4 and 89.1% in male and female WT mice, respectively, compared with non-fasted WT mice of the same sex and remained unchanged in fasted male CRF-OE mice. By contrast, female CRF-OE mice had 50.5% higher basal hypothalamic NPY mRNA level than WT females which was not changed after overnight fasting. Three-way ANOVA shows a significant influence by sex (*F*_1,30_ = 20.3, *P* < 0.001), feeding status (*F*_1,30_ = 18.3, *P* < 0.001), sex × genotype (*F*_1,30_ = 6.5, *P* = 0.016) and feeding status × genotype (*F*_1,30_ = 15.8, *P* < 0.001).

#### POMC mRNA (Fig. 6b)

The basal POMC expression was not significantly different in both male and female WT and CRF-OE mice. In males, an overnight fast decreased the POMC mRNA levels by 69.2 and 49.0% in WT and CRF-OE mice, respectively. By contrast, in females, fasting did not modify POMC mRNA levels in WT and increased it by 66.7% in CRF-OE mice. Three-way ANOVA displayed significant influence by sex (*F*_1,30_ = 28.3, *P* < 0.001), genotype (*F*_1,30_ = 11.4, *P* = 0.002), feeding status (*F*_1,30_ = 5.9, *P* = 0.021), and sex × feeding status (*F*_1,30_ = 17.9, *P* < 0.001).

#### Leptin receptor mRNA (Fig. 6c)

The basal and fasting levels of hypothalamic leptin receptor (LepR) mRNA were similar in male and female WT mice. In CRF-OE, basal LepR expression levels were increased significantly by 29.8 and 45.0% in male and female mice, respectively, compared with same sex WT mice and not significantly modified by fasting. Three-way ANOVA displayed significant influence only by genotype (*F*_1,30_ = 30.4, *P* < 0.001).

#### CRF_2_ receptor mRNA

The CRF_2_ transcript levels in the hypothalamus were not influenced by either fasting or non-fasting conditions or genotype (WT and CRF-OE) of the mice (data not shown).

#### Correlation between the hypothalamic signals with circulating hormones and perigonadal fat

The expression of NPY in the hypothalamus displayed no significant correlation with peripheral hormones when all mice were analyzed together (data not shown). In non-fasted male and female CRF-OE and WT mice, the NPY mRNA levels were correlated with those of LepR (*P* < 0.40) and to a smaller extent with plasma leptin (*P* < 0.25; Additional file 1: Figure S7A–B). In overnight fasted male and female CRF-OE and WT mice, NPY expression was correlated POMC mRNA levels and perigonadal fat (Additional file 1: Figure S7C, D). In male CRF-OE and WT mice under basal and fasting conditions, NPY expression was correlated with plasma ghrelin and negatively with leptin and perigonadal fat (Additional file 1: Figure S7E–G), which were not detected in female mice (data not shown).

The correlations between the hypothalamic POMC mRNA and plasma acyl ghrelin levels were found in different conditions, namely basal and fasting, all mice and males (Additional file 1: Figure S8A–D), while with LepR mRNA, the correlation was very high in fasted male and female CRF-OE and WT mice (Additional file 1: Figure S8E, F).

The hypothalamic LepR expression was correlated with plasma leptin levels in all mice under basal and fasting conditions (Additional file 1: Figure S9).

## Discussion

CRF transgenic mice with elevated CRF expression primarily occurring in the brain and related to enlargement of adrenals and hypercorticosteronemia [1–3], display increased plasma levels of insulin and leptin, low circulating acyl ghrelin, and upregulation of leptin receptor expression in the hypothalamus compared to WT littermates. After an overnight fast, CRF-OE mice have hyperglycemia, maintain elevated leptin plasma levels, show no rise in acyl ghrelin, and inhibit refeeding response compared to WT mice. Moreover, the sex differences in body weight, perigonadal fat, and leptin plasma with lower levels in WT no longer exist in CRF-OE mice. Additionally, the adaptive response to an overnight fast which showed sex differences in WT mice in the percentage of body weight change, perigonadal fat weight, food intake, and alterations of insulin, leptin, and ghrelin plasma levels are also lost in CRF-OE mice. However, unlike WT mice, CRF-OE mice display sex differences in the fasting hyperglycemia and hypothalamic NPY and POMC mRNA levels with females > males. These findings point to the impact of chronic CRF expression in the brain in altering aspects of adaption to metabolic stress and sex differences.

### Adrenal glands, corticosterone, and abdominal obesity

Chronic intracerebroventricular infusion of CRF in rats resulted in adrenal hypertrophy [30]. Likewise, under condition of CRF-OE expression primarily in specific brain nuclei [1, 2], the adrenal glands’ weight was significantly higher in CRF-OE than in WT mice and there was a sex difference (females > males) in both genotypes consistent with previous reports [3, 12, 31]. This enlargement in CRF-OE mice is related to the hypertrophy of the adrenal cortex, a zone known to be involved in the corticosteroid synthesis and secretion, while the adrenal medulla is not altered [3, 12, 31]. Consistent with these previous histological findings [3, 12, 31], there is a high correlation between the adrenal gland weights and levels of plasma corticosterone in male and female CRF-OE mice and WT littermates. Although there was no significant sex difference in corticosterone levels, the correlation indicates to some extent that enlarged glands lead to high corticosterone production.

CRF-OE male and female mice displayed abdominal obesity [3, 9, 22] as assessed in the present study by increased weights in perigonadal fat while body weights were similar to WT of both sexes. Other studies showed that the weights of perirenal and inguinal fat and the total body fat content were also increased when assessed either in male or female CRF transgenic mice [22, 31]. The hypertrophy of abdominal adipose tissue is known to occur with glucocorticoid excess [32–34]. Male and female CRF-OE mice have similar hypercorticosteronemia reaching 5.6- and 4.2-fold higher levels than WT mice, respectively, which is correlated with the epididymal weight. In addition, other studies showed that adrenalectomy completely reversed the increased epididymal and perirenal fat weight observed in CRF-OE mice [22], indicative that glucocorticoid excess is responsible for the hypertrophy of visceral adipose tissue. Visceral fat contains more glucocorticoid receptors than fat in the subcutaneous regions, which facilitates the development of abdominal obesity in addition to buffering the excess of glucocorticoids [34–37]. The loss of sex difference in body and perigonadal fat weight could be the consequences of sustained high levels of corticosterone [1, 3, 9, 22].

### Circulating the levels of insulin, leptin, and acyl ghrelin under basal and fasted conditions

Under basal conditions, we found that female WT mice displayed lower basal plasma leptin levels than males similar to other reports in rats [38, 39], while sex difference did not exist in CRF-OE mice. These disparities are likely to be related to the reduced amounts of fat mass in female WT compared to male WT mice while male and female CRF-OE mice have similarly increased fat weights. A previous report showed that male CRF-OE had elevated plasma insulin levels and hyperglycemia [22] consistent with our observations. In addition, we found that both leptin and insulin plasma levels were similarly increased in both male and female CRF-OE compared to WT. Circulating leptin and insulin are also detected to be elevated in other types of obesity such as induced by high-fat diet, idiopathic, and in patients with Cushing’s disease [40–42]. The high correlations between plasma leptin and insulin levels and body fat mass found in CRF-OE and WT mice also occurred in mice fed a high-fat diet or in obese humans [40, 43, 44]. In addition, there was a positive correlation between basal circulating levels of leptin and insulin with those of corticosterone in CRF-OE mice. Other reports showed that adrenalectomy completely prevented the hyperinsulinemia and hyperglycemia in male CRF-OE mice [22]. Glucocorticoids may directly act to stimulate leptin synthesis and release based on evidence that a half site of the glucocorticoid response element is present in the 5′-flanking region of the human leptin gene [45, 46], and leptin synthesis and secretion are increased by dexamethasone treatment in human or rat adipocytes [41, 47]. Moreover, adrenalectomy in rats, surgical treatment in Cushing’s patients, and weight loss in obese subjects are followed by the normalization of plasma leptin levels and an increase in leptin sensitivity which coincides with the reversal of glucocorticoid levels [41, 42, 48]. Collectively, these data support that glucocorticoids excess itself in CRF-OE is responsible for the elevated circulating levels of leptin and insulin and the hyperglycemia.

After an overnight fast, WT females did not show a significant reduction in plasma leptin and insulin as observed in male WT mice, which could be due to the already low basal levels of these hormones in females. CRF-OE showed a blunted reduction of leptin in both sexes by fasting which was also observed in diet-induced obesity (DIO) mice exposed to a prolonged fast (48 h) [40] and in Zucker (fa/fa) rats [49]. The significant correlation between leptin and insulin levels under fasting conditions in mice and humans [50] is consistent with that the two hormones are functionally related to energy homeostasis.

Basal plasma acyl ghrelin levels were lowered in both male and female CRF-OE mice compared to WT mice. The factors reducing plasma acyl ghrelin could be related to the volume of adipose tissues since lower ghrelin levels are found in different types of obesity [51–53]. It is well known that fasting reduces plasma levels of insulin and leptin while acyl ghrelin levels rise in lean rodents [49, 54–56] as also found in male WT mice (present study). However, fasting did not increase plasma levels of acyl ghrelin in male and female CRF-OE mice as well as in female WT mice contrasting with the overnight fasting-induced increase in plasma total ghrelin previously observed in both male WT and CRF-OE mice [18]. This may be indicative of an increased ratio of desacyl/acyl ghrelin reported to occur under stress conditions [57]. Another report also showed an impaired plasma acyl ghrelin response to fasting in genetically obese Zucker rats [58]. In contrast to CRF-OE mice, acyl ghrelin was increased in DIO mice with reduced hyperphagia after fasting which was considered as a stress response [59]. The blunted increase in acyl ghrelin release by fasting in CRF-OE mice could result from more complex mechanisms of imbalanced hormone levels. Namely, it is known that feeding is associated with central vagal activation that stimulates ghrelin release [60] and the overdrive of CRF in the brain inhibits the vagal preganlionic motor neurons in rats [61]. In the present study, it was unexpected that fasting acyl ghrelin levels were not increased in female WT littermates although they ingested more food per body weight compared to male WT mice. This provides the first evidence of a sex difference in the acyl ghrelin response to an overnight fast in mice. This is unlikely to be related to differences in blood glucose levels or hypercorticosteronemia found to be similar in male and female WT mice after fasting. More studies are warranted to ascertain this sex difference in other rodent species and whether somatostatin acting on somatostatin 2 receptors located on gastric ghrelin cells plays a role [62] or gonadal steroid hormones are involved [63]. However, these data indicate that the increased feeding response to a fast in female compared to male WT mice is not directly related to changes in circulating acyl ghrelin levels.

The similar plasma levels of PYY and PP in CRF-OE and WT mice under basal or fasted conditions are indicative that these two hormones do not play a role in the differential feeding response to a fast in a model of abdominal obesity with chronic HPA stimulation which is consistent with rodent models of DIO [64]. The high correlation of PYY and PP levels under basal, unlike fasted conditions, indicates that PYY and PP, released from the same gut endocrine cells, are functionally related [65, 66].

### Alterations in the hypothalamic signals under basal and fasted conditions

In the brain, NPY and POMC neurons in the arcuate nucleus are key players in regulating energy intake [19, 20]. Signals are mediated via integrating converging peripheral signals including acyl ghrelin, leptin, and insulin [67]. We previously reported that male CRF-OE mice exposed to an overnight fast had a decreased neuronal activation in arcuate nucleus neurons as shown by the reduced number of Fos-positive neurons compared to WT mice [18]. We previously reported that peripheral administration of acyl ghrelin-induced activation of arcuate nucleus neurons occurred in NPY-synthesizing neurons in mice [68]. It is also well established that fasting increases hypothalamic NPY mRNA levels [56, 69]. In the present study, NPY mRNA levels increased similarly in overnight fasted male and female WT mice while such a rise was not observed in CRF-OE mice. By contrast, the suppression of hypothalamic POMC mRNA levels occurring after a fast in mice [70] was similar in WT and CRF-OE male mice. These data point to the altered peripheral acyl ghrelin and hypothalamic NPY responses to a fast in male CRF-OE male mice as potentially playing a role in the blunted refeeding food intake response after an overnight fast. Noticeably, there was a sex difference in the hypothalamic response to fasting as showed by the rise in POMC mRNA expression in female CRF-OE while NPY levels were not modified. However, both male and female CRF-OE mice had similar inhibition of feeding response to the fast, suggesting that the increased POMC anorexic signaling may play a more prominent role in female CRF-OE. The lack of NPY mRNA rise in the hypothalamus induced by fasting in female CRF-OE mice may be related to the already elevated basal level which is similar to that induced by refeeding in WT mice. Other studies in mice showed that chronic elevation of NPY overexpression does not induce changes in feeding behavior [71], and there is evidence that chronic stress-induced reduction of food intake in rodents is associated with elevated expression of NPY in the arcuate nucleus [72]. It can be speculated that the elevation of NPY expression in the arcuate nucleus observed selectively in female CRF-OE mice reflects a counter regulatory anxiolytic mechanism [72] to reverse enhanced sensitivity of female to brain CRF overexpression [11] and induction of anxiety [7].

We also observed an increased leptin receptor levels in CRF-OE mice which may be related to possible leptin resistance due to truncal obesity, as shown by the correlation between leptin receptor expression and circulating leptin levels. The observed sex differences in NPY and POMC mRNA in CRF-OE mice imply that sex hormones signaling in the brain modulate the hypothalamic circuits of NPY and POMC. The underlying mechanisms warrant further investigation.

## Conclusions

CRF-OE mice, known as a model for chronic stress and Cushing’s disease, display altered circulating endocrine hormones related to energy balance as shown by the elevated plasma levels of leptin and insulin and decreased acyl ghrelin levels associated with adrenal hypertrophy, hypercorticosteronemia, and increased abdominal fat. CRF-OE mice lose the sex difference in food intake and altered metabolic hormone responses to an overnight fast observed in WT mice (Table 3). This may be related to the increased corticosterone, insulin and leptin levels, and visceral fat volume similarly elevated in male and female CRF-OE mice. The sex difference in the alterations of hypothalamic NPY and POMC expression in response to fasting may be an indicative of the differential mechanisms involved in the inhibition of refeeding between male and female CRF-OE mice. Whether this reflects a potential influence of gonadal steroid hormones interaction with CRF signaling in the brain needs to be further investigated. In addition, the higher adrenal gland volume [3, 12, 31] and blood glucose levels in female than male CRF-OE mice may be consistent with the elevated locus coeruleus neuronal activity selectively occurring in female CRF-OE mice compared to males driving sympathetic outflow [12].

**Table 3:** Summary of alterations in CRF-OE mice and WT littermates influenced by CRF-OE and fasting with sex difference

		Basal		Fasting	
		WT	CRF-OE	WT	CRF-OE
Body weight		F < M	F↑	↓ F > M	↓ < WT
Fat		F < M	↑	↓ F < M	(−)
Adrenal glands		F > M	↑ F > M	(−)	(−)
Fasting blood glucose				(−)	↑ F > M
Refeeding				↑ F > M	↓
Plasma	Corticosterone	(−)	↑	↑	(−)
	Leptin	F < M	↑	M↓	(−)
	Insulin	(−)	↑	↓	↓
	Acyl ghrelin	(−)	↓	M↑	(−)
	PYY	(−)	(−)	M↓	M↓
Hypothalamus	NPY	(−)	F↑	↑	(−)
	POMC	(−)	(−)	M↓	M↓, F↑
	LepR	(−)	↑	(-)	(-)

## Additional file

Additional file 1: Figure S1. Plasma PP in male and female fasted and non-fasted (NF) CRF-OE and WT mice. **Figure S2.** Correlations of basal plasma levels of corticosterone and insulin (A), leptin (B), and acyl ghrelin (C) in CRFOE and WT mice. **Figure S3.** Correlations of basal plasma levels between PYY and PP (A), leptin and insulin (B), acyl ghrelin and insulin (C), and acyl ghrelin and leptin (D) in CRF-OE and WT mice. **Figure S4.** Correlation of plasma leptin and insulin in fasting CRF-OE and WT mice. **Figure S5.** Correlations of basal plasma leptin and insulin (A) perigonadal fat with leptin (B), insulin (C), and acyl ghrelin (D) in CRF-OE and WT mice under basal conditions. **Figure S6.** Correlations of perigonadal fat and leptin (A), insulin (B), or acylghrelin (C) in fasting CRF-OE and WT mice. **Figure S7.** Correlations of hypothalamic NPY expression with other signals or hormones in CRF-OE and WT mice: basal levels with leptin receptor mRNA (A) and plasma leptin (B), fasting levels with POMC mRNA (C) and perigonadal fat (D), basal and fasting levels in male mice with plasma acyl ghrelin (E) and leptin (F), and with perigonadal fat (G). **Figure S8.** Correlations of hypothalamic POMC expression with other signals or hormones in CRF-OE and WT mice: basal and fasting levels with plasma acyl ghrelin in all mice (A), basal and fasting levels with plasma acyl ghrelin in male CRF-OE and WT mice (B), basal levels with plasma acyl ghrelin in male CRF-OE and WT mice (C), fasting levels with plasma acyl ghrelin in male CRF-OE and WT mice (D), basal levels with leptin receptor mRNA in female CRF-OE and WT mice (E), and fasting levels with leptin receptor mRNA in CRF-OE and WT mice (F). **Figure S9.** Correlations between hypothalamic leptin receptor expression and plasma leptin in CRF-OE and WT mice. (PDF 103 kb)

## Abbreviations

ACTH: Adrenocorticotropic hormone
Acyl: Acylated
ANOVA: Analysis of variance
BW: Body weight
CRF: Corticotropin-releasing factor
CRF_2_: CRF receptor 2
HPA: Hypothalamic-pituitary-adrenal axis
LepR: Leptin receptor
NPY: Neuropeptide Y
OE: Overexpressing or overexpression
POMC: Proopiomelanocortin
PP: Pancreatic polypeptide
PYY: Peptide tyrosine tyrosine
WT: Wild-type
