# Supplementary material for: Corticotropin-releasing factor overexpression in mice abrogates sex differences in body weight, visceral fat, and food intake response to a fast and alters levels of feeding regulatory hormones
Source: Biol Sex Differ. 2017 Jan 13;8:2. doi: 10.1186/s13293-016-0122-6 (PMC5237138; doi:10.1186/s13293-016-0122-6)

Figure S1

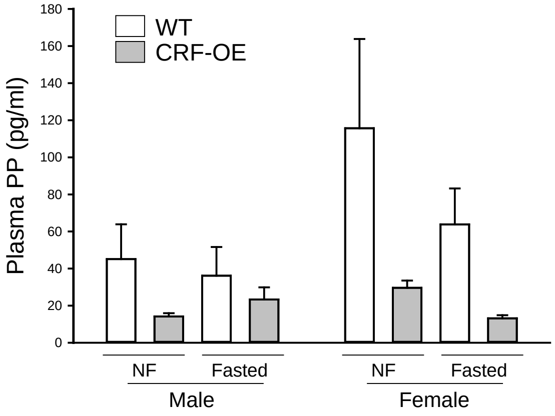
180
160
140
120
100
80
60
40
20
0
Plasma PP (pg/ml)
WT
CRF-OE
NF
Fasted
Male
NF
Fasted
Female

Figure S2

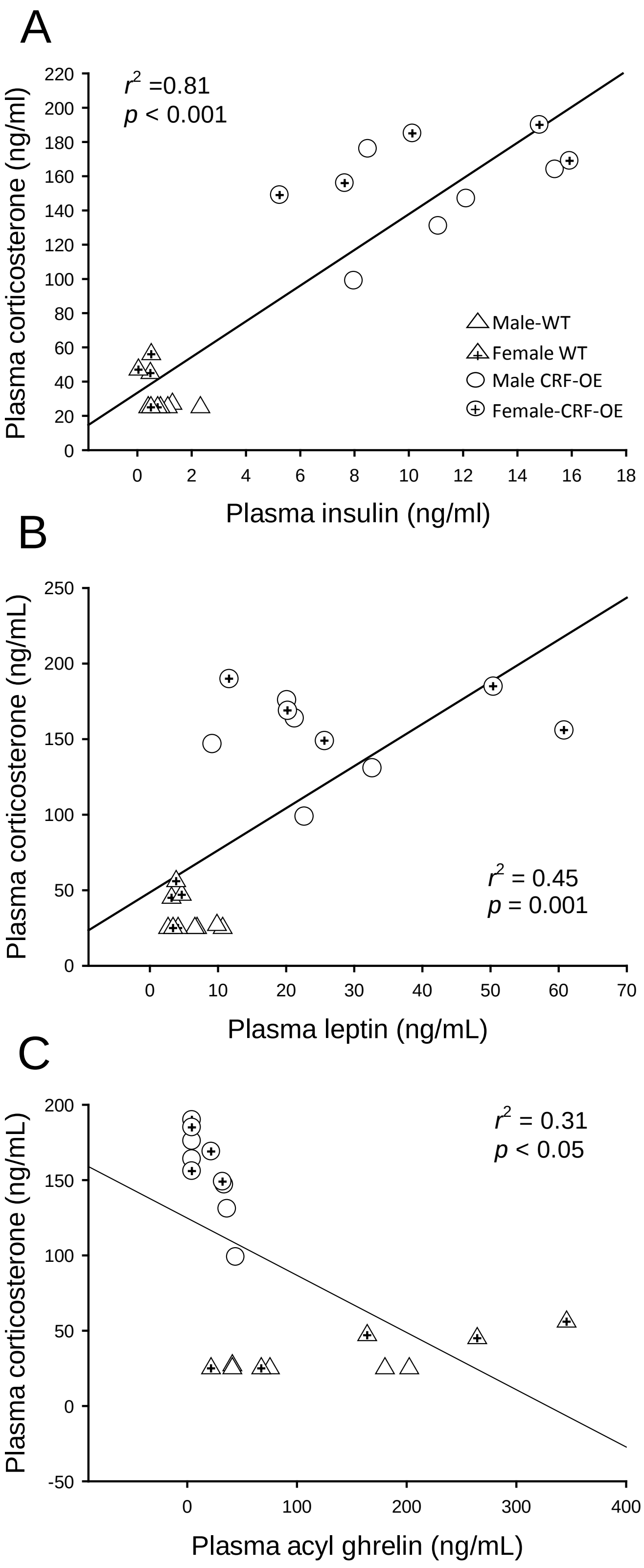
A
r^2 =0.81
p < 0.001
Plasma corticosterone (ng/ml)
Plasma insulin (ng/ml)
Male-WT
Female WT
Male CRF-OE
Female-CRF-OE
B
r^2 = 0.45
p = 0.001
Plasma corticosterone (ng/mL)
Plasma leptin (ng/mL)
C
r^2 = 0.31
p < 0.05
Plasma corticosterone (ng/mL)
Plasma acyl ghrelin (ng/mL)

Figure S3

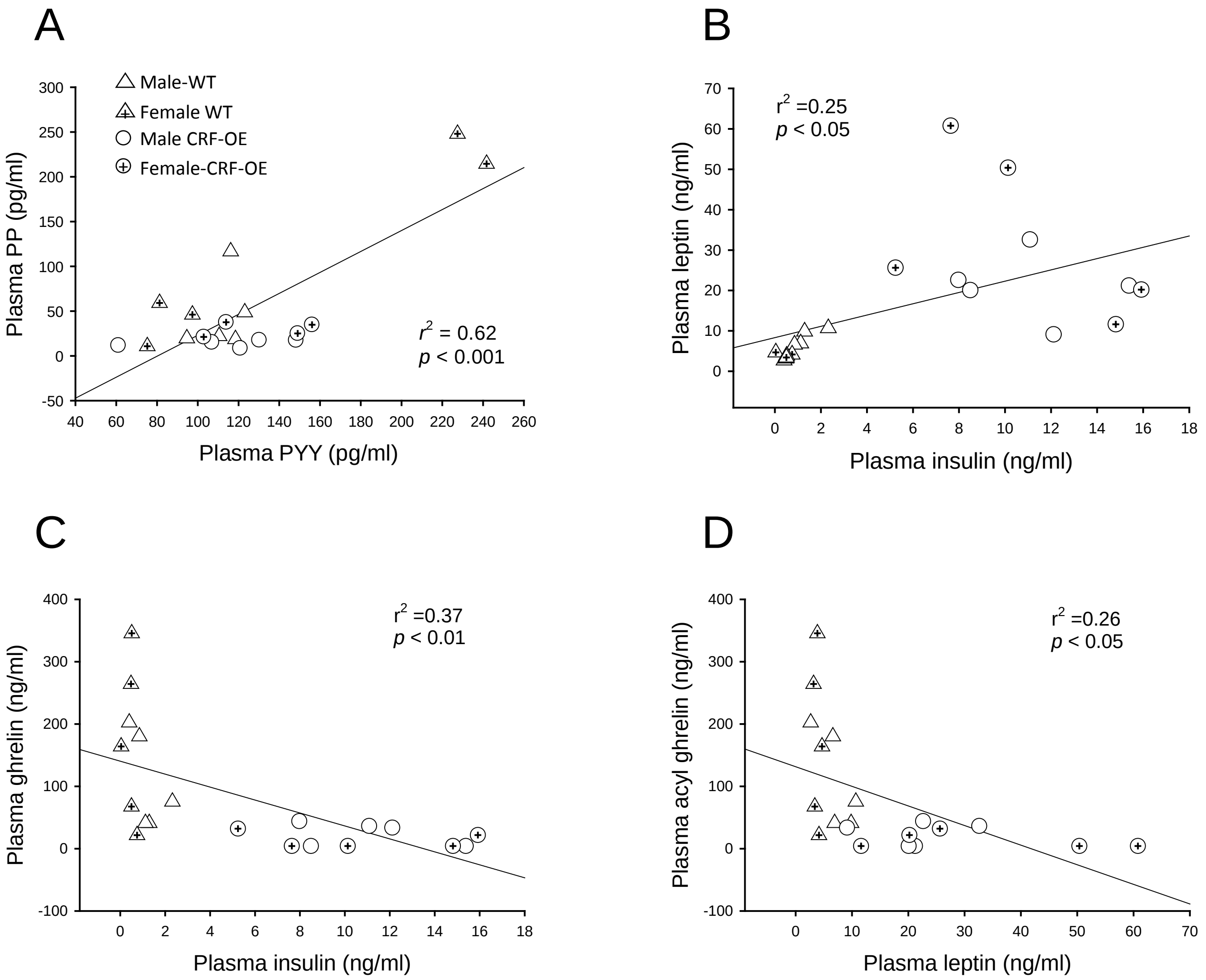
A
B
C
D
Male-WT
Female WT
Male CRF-OE
Female-CRF-OE
Plasma PP (pg/ml)
Plasma PYY (pg/ml)
r^2 = 0.62
p < 0.001
Plasma leptin (ng/ml)
Plasma insulin (ng/ml)
r^2 =0.25
p < 0.05
Plasma ghrelin (ng/ml)
Plasma insulin (ng/ml)
r^2 =0.37
p < 0.01
Plasma acyl ghrelin (ng/ml)
Plasma leptin (ng/ml)
r^2 =0.26
p < 0.05

Figure S4

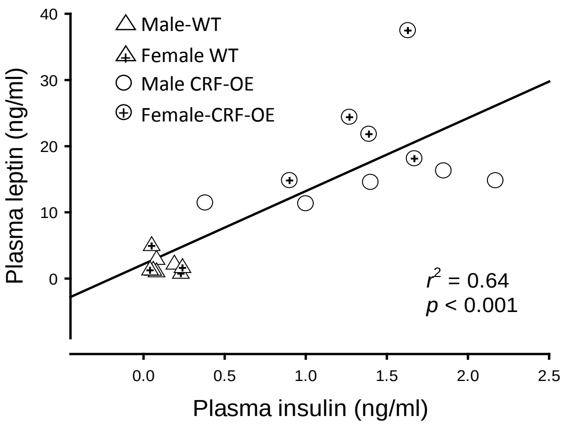
Male-WT
Female WT
Male CRF-OE
Female-CRF-OE
Plasma leptin (ng/ml)
40
30
20
10
0
0.0
0.5
1.0
1.5
2.0
2.5
Plasma insulin (ng/ml)
$r^2 = 0.64$
$p < 0.001$

Figure S5

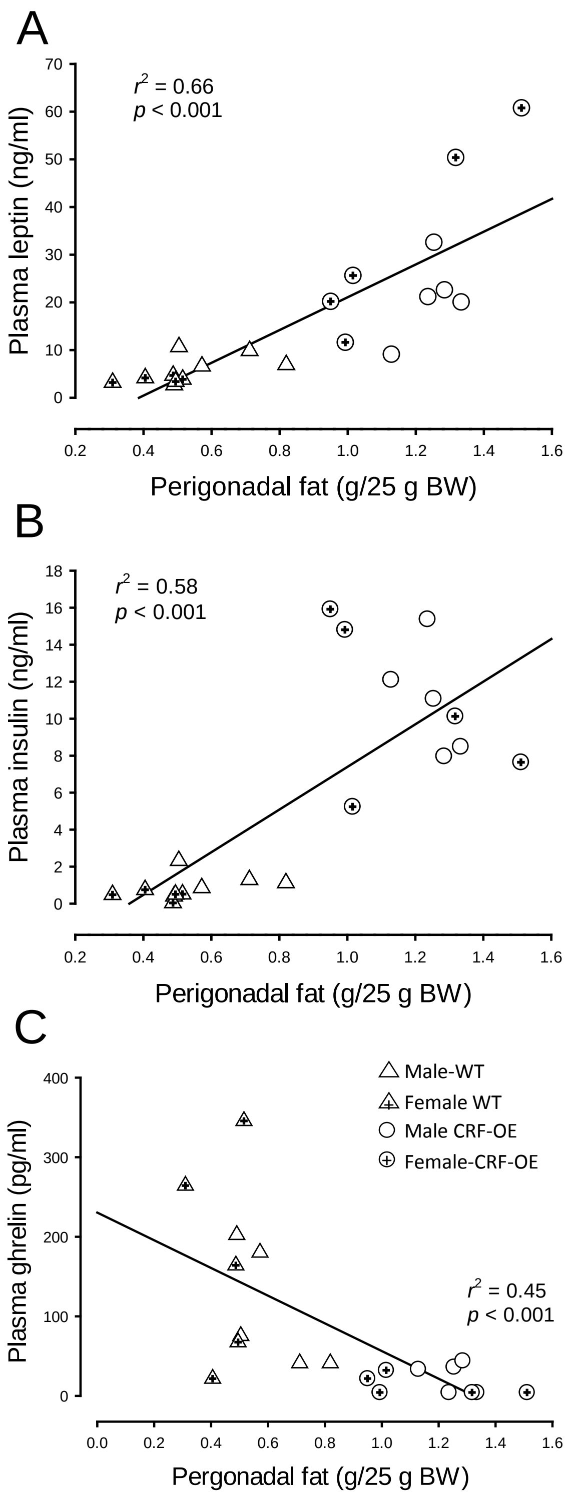
A
70
60
50
40
30
20
10
0
r^2 = 0.66
p < 0.001
Plasma leptin (ng/ml)
0.2
0.4
0.6
0.8
1.0
1.2
1.4
1.6
Perigonadal fat (g/25 g BW)
B
18
16
14
12
10
8
6
4
2
0
r^2 = 0.58
p < 0.001
Plasma insulin (ng/ml)
0.2
0.4
0.6
0.8
1.0
1.2
1.4
1.6
Perigonadal fat (g/25 g BW)
C
400
300
200
100
0
Male-WT
Female WT
Male CRF-OE
Female-CRF-OE
r^2 = 0.45
p < 0.001
Plasma ghrelin (pg/ml)
0.0
0.2
0.4
0.6
0.8
1.0
1.2
1.4
1.6
Pergonadal fat (g/25 g BW)

Figure S6

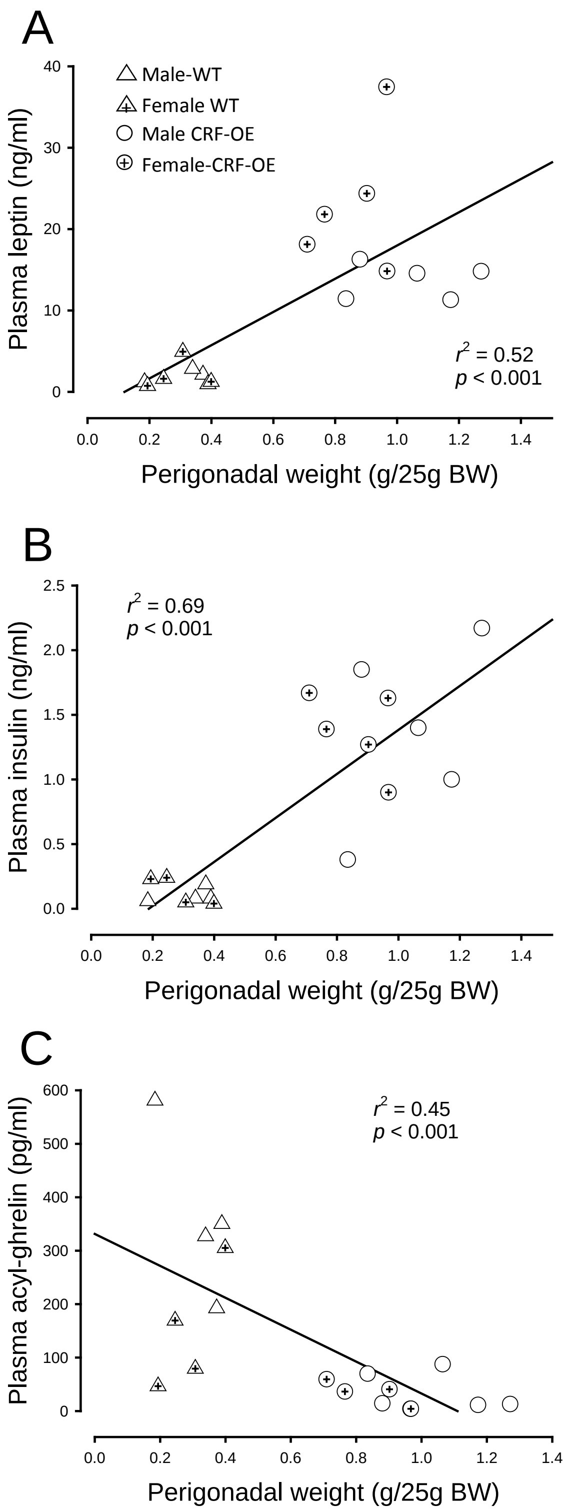
A
Male-WT
Female WT
Male CRF-OE
Female-CRF-OE
Plasma leptin (ng/ml)
r2 = 0.52
p < 0.001
Perigonadal weight (g/25g BW)
B
Plasma insulin (ng/ml)
r2 = 0.69
p < 0.001
Perigonadal weight (g/25g BW)
C
Plasma acyl-ghrelin (pg/ml)
r2 = 0.45
p < 0.001
Perigonadal weight (g/25g BW)

Figure S7

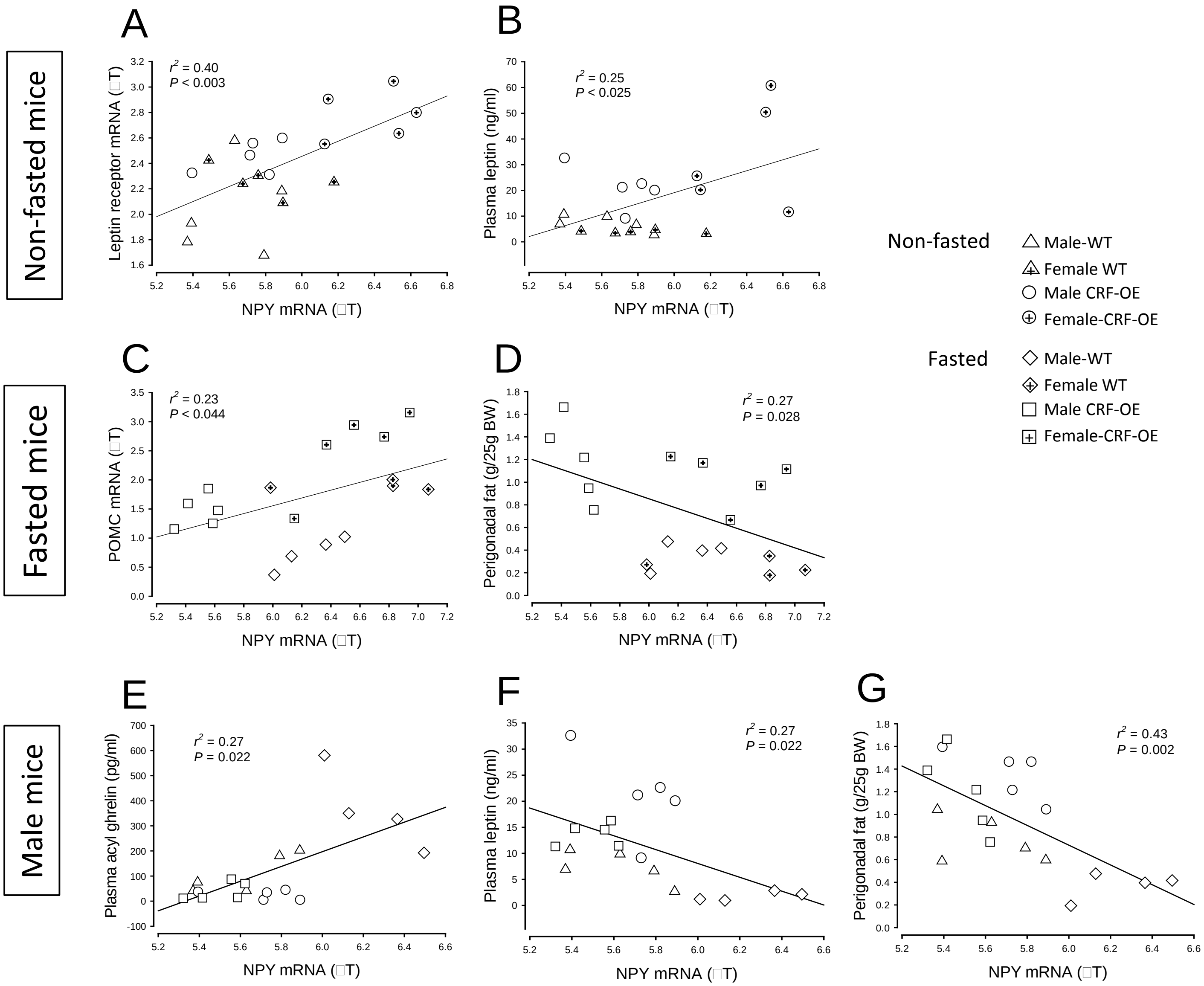
A
B
C
D
E
F
G
Non-fasted mice
Fasted mice
Male mice
r^2 = 0.40
P < 0.003
Leptin receptor mRNA (□T)
NPY mRNA (□T)
r^2 = 0.25
P < 0.025
Plasma leptin (ng/ml)
r^2 = 0.23
P < 0.044
POMC mRNA (□T)
r^2 = 0.27
P = 0.028
Perigonadal fat (g/25g BW)
r^2 = 0.27
P = 0.022
Plasma acyl ghrelin (pg/ml)
r^2 = 0.27
P = 0.022
Plasma leptin (ng/ml)
r^2 = 0.43
P = 0.002
Perigonadal fat (g/25g BW)
Non-fasted
Male-WT
Female WT
Male CRF-OE
Female-CRF-OE
Fasted
Male-WT
Female WT
Male CRF-OE
Female-CRF-OE

Figure S8

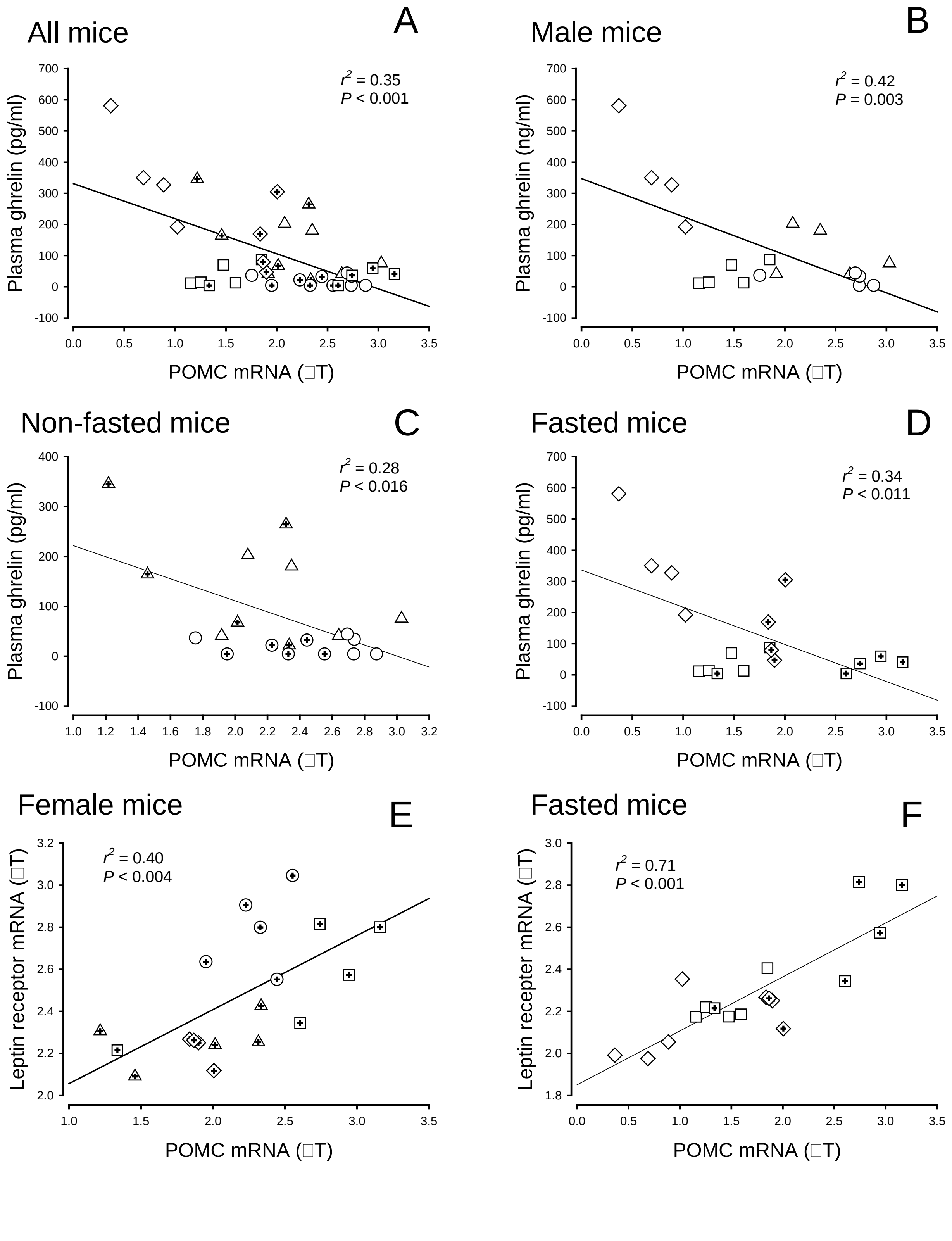
All mice
A
r^2 = 0.35
P < 0.001
Plasma ghrelin (pg/ml)
POMC mRNA (□T)
Male mice
B
r^2 = 0.42
P = 0.003
Plasma ghrelin (ng/ml)
POMC mRNA (□T)
Non-fasted mice
C
r^2 = 0.28
P < 0.016
Plasma ghrelin (pg/ml)
POMC mRNA (□T)
Fasted mice
D
r^2 = 0.34
P < 0.011
Plasma ghrelin (pg/ml)
POMC mRNA (□T)
Female mice
E
r^2 = 0.40
P < 0.004
Leptin receptor mRNA (□T)
POMC mRNA (□T)
Fasted mice
F
r^2 = 0.71
P < 0.001
Leptin recepter mRNA (□T)
POMC mRNA (□T)

Figure S9

All mice
A

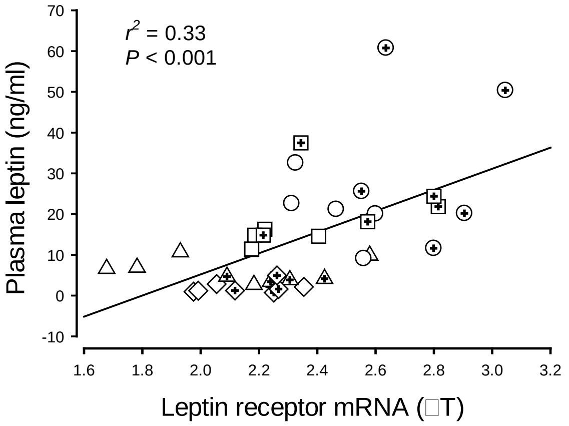

Non-fasted mice
B

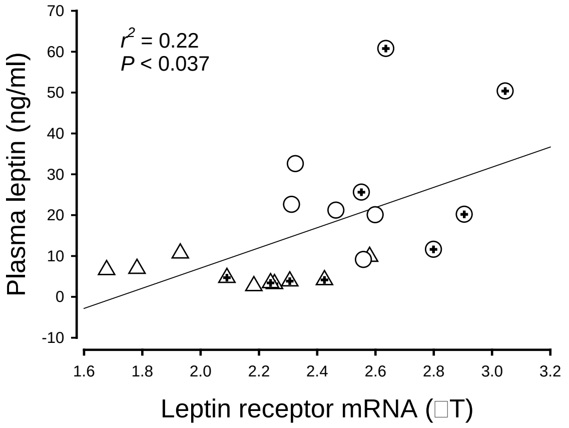

Fasted mice
C

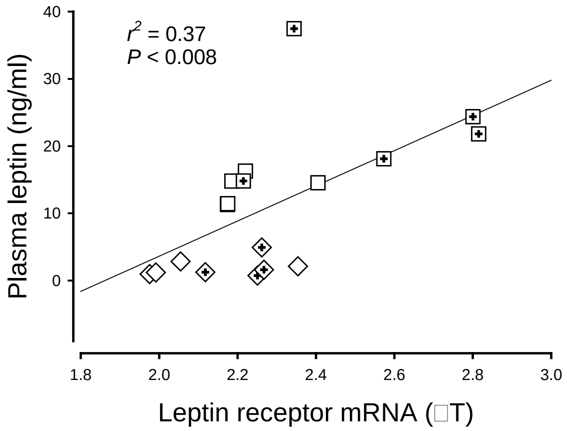

Supplement: Additional file 1: Figure S1. — Plasma PP in male and female fasted and non-fasted (NF) CRF-OE and WT mice. Figure S2. Correlations of basal plasma levels of corticosterone and insulin (A), leptin (B), and acyl ghrelin (C) in CRFOE and WT mice. Figure S3. Correlations of basal plasma levels between PYY and PP (A), leptin and insulin (B), acyl ghrelin and insulin (C), and acyl ghrelin and leptin (D) in CRF-OE and WT mice. Figure S4. Correlation of plasma leptin and insulin in fasting CRF-OE and WT mice. Figure S5. Correlations of basal plasma leptin and insulin (A) perigonadal fat with leptin (B), insulin (C), and acyl ghrelin (D) in CRF-OE and WT mice under basal conditions. Figure S6. Correlations of perigonadal fat and leptin (A), insulin (B), or acylghrelin (C) in fasting CRF-OE and WT mice. Figure S7. Correlations of hypothalamic NPY expression with other signals or hormones in CRF-OE and WT mice: basal levels with leptin receptor mRNA (A) and plasma leptin (B), fasting levels with POMC mRNA (C) and perigonadal fat (D), basal and fasting levels in male mice with plasma acyl ghrelin (E) and leptin (F), and with perigonadal fat (G). Figure S8. Correlations of hypothalamic POMC expression with other signals or hormones in CRF-OE and WT mice: basal and fasting levels with plasma acyl ghrelin in all mice (A), basal and fasting levels with plasma acyl ghrelin in male CRF-OE and WT mice (B), basal levels with plasma acyl ghrelin in male CRF-OE and WT mice (C), fasting levels with plasma acyl ghrelin in male CRF-OE and WT mice (D), basal levels with leptin receptor mRNA in female CRF-OE and WT mice (E), and fasting levels with leptin receptor mRNA in CRF-OE and WT mice (F). Figure S9. Correlations between hypothalamic leptin receptor expression and plasma leptin in CRF-OE and WT mice. (PDF 103 kb) [file 13293_2016_122_MOESM1_ESM.pdf]
